# Completeness of Digital Accessible Knowledge (DAK) about terrestrial mammals in the Iberian Peninsula

**DOI:** 10.1371/journal.pone.0213542

**Published:** 2019-03-08

**Authors:** Nora Escribano, David Galicia, Arturo Hugo Ariño

**Affiliations:** Universidad de Navarra, Department of Environmental Biology, Pamplona, Spain; USDA Forest Service, UNITED STATES

## Abstract

The advent of online data aggregator infrastructures has facilitated the accumulation of Digital Accessible Knowledge (DAK) about biodiversity. Despite the vast amount of freely available data records, their usefulness for research depends on completeness of each body of data regarding their spatial, temporal and taxonomic coverage. In this paper, we assess the completeness of DAK about terrestrial mammals distributed across the Iberian Peninsula. We compiled a dataset with all records about mammals occurring in the Iberian Peninsula available in the Global Biodiversity Information Facility and in the national atlases from Portugal and Spain. After cleaning the dataset of errors as well as records lacking collection dates or not determined to species level, we assigned all occurrences to a 10-km grid. We assessed inventory completeness by calculating the ratio between observed and expected richness (based on the Chao2 richness index) in each grid cell and classified cells as well-sampled or under-sampled. We evaluated survey coverage of well-sampled cells along four environmental gradients and temporal coverage. Out of 796,283 retrieved records, quality issues led us to remove 616,141 records unfit for this use. The main reason for discarding records was missing collection dates. Only 25.95% cells contained enough records to robustly estimate completeness. The DAK about terrestrial mammals from the Iberian Peninsula was low, and spatially and temporally biased. Out of 5,874 cells holding data, only 620 (9.95%) were classified as well-sampled. Moreover, well-sampled cells were geographically aggregated and reached inventory completeness over the same temporal range. Despite the increasing availability of DAK, its usefulness is still compromised by quality issues and gaps in data. Future work should therefore focus on increasing data quality, in addition to mobilizing unpublished data.

## Introduction

The mobilization via the Internet of a vast amount of biodiversity data offers new opportunities for basic research and evidence-based decision-making in conservation [1–3]. Biodiversity data exchange infrastructures such as the Global Biodiversity Information Facility (GBIF) facilitate access to massive amounts of primary biodiversity data records (PBR) which become digital accessible knowledge (DAK [4]; also referred to as digital accessible information or DAI [5]). However, the unprecedented availability of digital PBR still seems insufficient to overcome existing biodiversity information gaps [5–7]. Data portals usually aggregate smaller datasets derived from local surveys designed for a specific purpose. GBIF, the largest portal, enabled access to more than one billion (10^9^) PBR in 40,521 datasets as of August 2018. Taxonomic, spatial and temporal gaps arise inevitably from the nature of these data aggregators [8].

Ensuring a degree of completeness of biodiversity databases is fundamental to obtaining reliable results to map species richness [9,10] or to improve the design of sampling campaigns [5,8,11]. Recently, researchers have engaged in developing methods to determine inventory completeness and in providing guidelines to assess gaps in biodiversity data [12]. Much of this research focused on botanical databases in megadiverse areas [5,13–16], although other taxonomic groups and areas have also been explored [8,17,18].

Records are not free from uncertainties and errors. The quality of biodiversity data shared online has been long questioned in the literature. Spatial and sampling effort biases have been discussed frequently [7,19,20]. There is thus a baseline of literature about how to quantify these spatial errors and how to deal with their effects. However, temporal gaps and issues seem to have been addressed much less often [5,14,21,22], even though time is one of the key attributes of a PBR: where and *when* a specimen *was* recorded [23]. Such an oversight might perhaps owe to the contemporary nature of most DAK [24].

The Iberian Peninsula constitutes an interesting biogeographical area, as it is one of the important Pleistocene glacial refugia [25], influenced by both the Atlantic Ocean and the Mediterranean Sea. Its wide range of climates, and its complex variety of landscapes and habitats, have a positive effect on biodiversity [26]. Regarding the mammal community, the Iberian Peninsula is home to typically Central European species, which find their southern distribution limit within the peninsula, while it also hosts Mediterranean species, many of them as endemic species [27].

The purpose of this study was to determine the completeness of DAK about terrestrial mammals in the Iberian Peninsula. We focused on both spatial and temporal gaps existing in the digital accessible knowledge [4] as represented by the data available through GBIF and the data in the national atlases of Spain [27] and Portugal [28]. We also assessed how the dataset covered the environmental characteristics of the territory. The analysis was done on the entire dataset and then repeated after splitting the dataset by taxonomic groups [29] to test whether the inventory completeness and survey and temporal coverage were taxonomically dependent.

## Material and methods

### Study area

The Iberian Peninsula is the westernmost peninsula of Europe. Its location facing both the Atlantic Ocean and the Mediterranean Sea, while almost touching the African continent, provides for a broad range of climates. Topography also contributes to its variety of habitats and landscapes that include temperate and Mediterranean forests, and scrublands [26]. Human activity has had a significant effect on the peninsula, modifying its territory towards a predominately agricultural landscape [30].

### Biodiversity data

We downloaded from GBIF all georeferenced records from the Iberian Peninsula that met the taxonkey ‘Mammalia’ in October 2016 for a preliminary analysis and proof of concept (http://doi.org/10.15468/dl.mcbdbq and http://doi.org/10.15468/dl.exhvrs2) and then an updated dataset in September 2018 (https://doi.org/10.15468/dl.acitdv) for the final analysis described here. The dataset was checked, cleaned and filtered in several steps. We first excluded records from the islands and Spanish cities in North Africa, domestic species and invasive species (e.g., raccoon and coypú). Secondly, we removed all the records that lacked a collection date, and that had not been determined down to species level. We also removed records that had coordinates falling outside the boundaries of the Iberian Peninsula. Thirdly, following Sousa-Baena et al. [4], we kept the unique combinations of 1) scientific name, 2) latitude and longitude, and 3) collection date.

We obtained from the Spanish Society of Mammalogists (SECEM) the raw data of the Spanish national atlas updated until 2016 [27], and downloaded the data of the Atlas of Portugal [28]. We repeated the GBIF cleaning process on these records (hereinafter “atlas data”). The information about collection date in the Atlas of Portugal was of very low resolution (i.e., records dated before or after 2000). Thus, we decided to use records from Portuguese atlas for the spatial gap assessment but omit them while addressing the temporal gaps.

While filtering the GBIF data records, we detected a remarkable peak of 179,757 (30.9%) observations in 2007. All of them belonged to one single dataset (ARM dataset) shared by the National Inventory of Terrestrial Species of Spain, which compiled information about terrestrial species in the country harvested from the Spanish national atlases of birds, amphibians, and mammals. These observations qualified as duplicates after merging the data in the Spanish atlas, even though the information level was different (more precise and exhaustive in the atlas data than in the version shared through GBIF). We removed those records as superseded by the atlas data which were also more up to date than the GBIF data.

Before merging both datasets, we assigned all records to the 10 km reference Universal Transverse Mercator (UTM) grid, corresponding to the lowest spatial resolution of the data we had, tagging each record with its cell’s ID. Joining both datasets may have resulted in the creation of duplicate records (same observation record uploaded twice), as the national atlases were built by contributions from different authors or institutions (e.g., natural history collections, research projects, opportunistic observations) who could also have independently uploaded their records to GBIF. To address this, we generated a marker for each record by combining scientific name, collection year and cell’s ID. Then, we used these markers to extract the unique records in the atlas dataset. We added the unique records to the GBIF dataset, thus building the full dataset.

We performed a factor correspondence analysis on the full dataset in order to observe taxonomical patterns or biases that could affect the results as a consequence of the difference in sampling methods [29]. A bimodal pattern was observed (Fig A in S1 Appendix), roughly corresponding to taxon groups of different size and sampling method: large- and medium-size mammals, usually detected by indirect methods such as footprints, camera trapping, or scats [31], and small mammals sampled through analysis of owl pellets or traps [32]. Accordingly, we segmented the data into two taxonomic groups: *non-small mammals* comprising the orders Lagomorpha, Artiodactyla, Carnivora, Chiroptera and the family Erinaceidae of the order Eulipotyphla, and *small mammals* including the order Rodentia and the family Soricidae of the order Eulipotyphla.

### Completeness analysis

We estimated inventory completeness based on the approaches of Sousa-Baena et al. [4] and Yang et al. [15]. For each cell, we calculated the expected number of species using the Chao2 index and completeness (C_c_) as the ratio between observed and expected richness, S_obs_/S_exp_ [33]. We also calculated the mean slope (C_m_) of the final 10% of the species accumulation curve [15]. We calculated these two estimators for the full dataset and for each taxonomic group.

The C_c_ index ranged from zero (low completeness) to one (high completeness). Cells with low sample levels can exhibit high but artificial completeness values [4]. Therefore, we established a minimum number of records per cell to calculate the estimators. This minimum was established by assessing the relationship between C_c_ and number of records, following Sousa-Baena et al. [4]. For the generation of the species accumulation curves (SAC), we used the function ‘speccacum’ (method = ‘exact’) in the R package *vegan* [34], and then we obtained the slope of the final 10% of the SAC. Values close to zero (flat slopes) indicated high completeness (saturation of the curve almost reached) whereas higher values (steeper slopes) indicated low completeness [15]. We plotted the completeness values based on C_c_ vs. C_m_ to test whether they were correlated. As the analysis showed high correlation for the full dataset (r Spearman: -0.60, p value < 0.001, Fig B in S1 Appendix) we chose to use C_c_. Finally, we classified the cells as well-sampled or under-sampled based according to two criteria, strict (high-threshold) and lax (low-threshold), based on thresholds used in previous studies but adapted to data availability [17]. Well-sampled cells for the strict criterion required having more than 50 records and a value of C_c_ equal to or greater than 0.8, while the lax criterion identified well-sampled cells as having more than 25 records and a value of C_c_ equal to or greater than 0.7. The following analyses were performed based on well-sampled cells according to the lax criterion.

### Environmental coverage

We evaluated the coverage of well-sampled cells along environmental gradients [17]. We downloaded bioclimatic variables at 2.5-minute resolution (approximately five km resolution) from the WorldClim database [35] and land cover data from the 2006 Corine Land Cover (CLC) database at 100-meter resolution from the European Environmental Agency [36].

Each climatic variable was averaged over each cell. For land cover uses, we first reclassified CLC categories into five new categories. CLC classifies land cover uses into 44 classes, grouped in a three-level hierarchy [36]. ‘Urban,’ ‘crops’ and ‘wetlands’ categories consisted of all land use categories grouped under classes 1, 2 and 4 respectively from the level 1 of the CLC nomenclature (“major categories”). Class 3 in the CLC nomenclature (‘Forest and semi-natural areas) was divided into two categories: ‘Forest’ included class 3.1 (Forests), and ‘scrubland’ included classes 3.2 and 3.3 (Shrub and herbaceous vegetation association and Open spaces with little or no vegetation, respectively). We then summarised the percentage of coverage of each land cover type in each cell.

We conducted a Variance Inflation Factor (VIF) analysis to discard correlated variables (VIF > 5), and correspondingly retained Annual Mean Temperature (AT), Annual Precipitation (AP), forest coverage (FC), and crops coverage (CC). Then we performed Kolmogorov-Smirnov (K-S) goodness-of-fit tests to compare the frequency distribution of well-sampled (lax criterion) and all sampled cells against the background cells (i.e. all territory) across the environmental gradients, following Clifford et al. [37]. Low values of the D statistic indicated that well-sampled cells reached a high level of survey coverage spanning all of the background environmental gradients [17].

### Temporal coverage

For each cell, we calculated (1) the median of collection years, (2) time since the last records were collected and (3) the interquartile range. Furthermore, we correlated C_c_ of well-sampled cells (lax criterion) with the median collection year to determine whether the high level of completeness was acquired from recent or historical surveys [14]. We also assessed whether well-sampled cells were spatially aggregated with regard to the decades in which completeness was reached using the Moran I test. Following Stropp [14], we calculated C_c_ for each cumulative decadal period, starting from 1900 to 1910, 1900 to 1920, …, until the last period 1900 to 2020. We assumed that a variation of 5% (upwards or downwards) of the final value of C_c_ was enough to conclude that the inventory completeness had been reached.

All analysis and data management were performed in R [38], and for the final graph modifications we used Inkscape [39]. See List A in S1 Appendix for references of the R packages employed in the study.

## Results

### Inventory completeness

**Fig 1:**
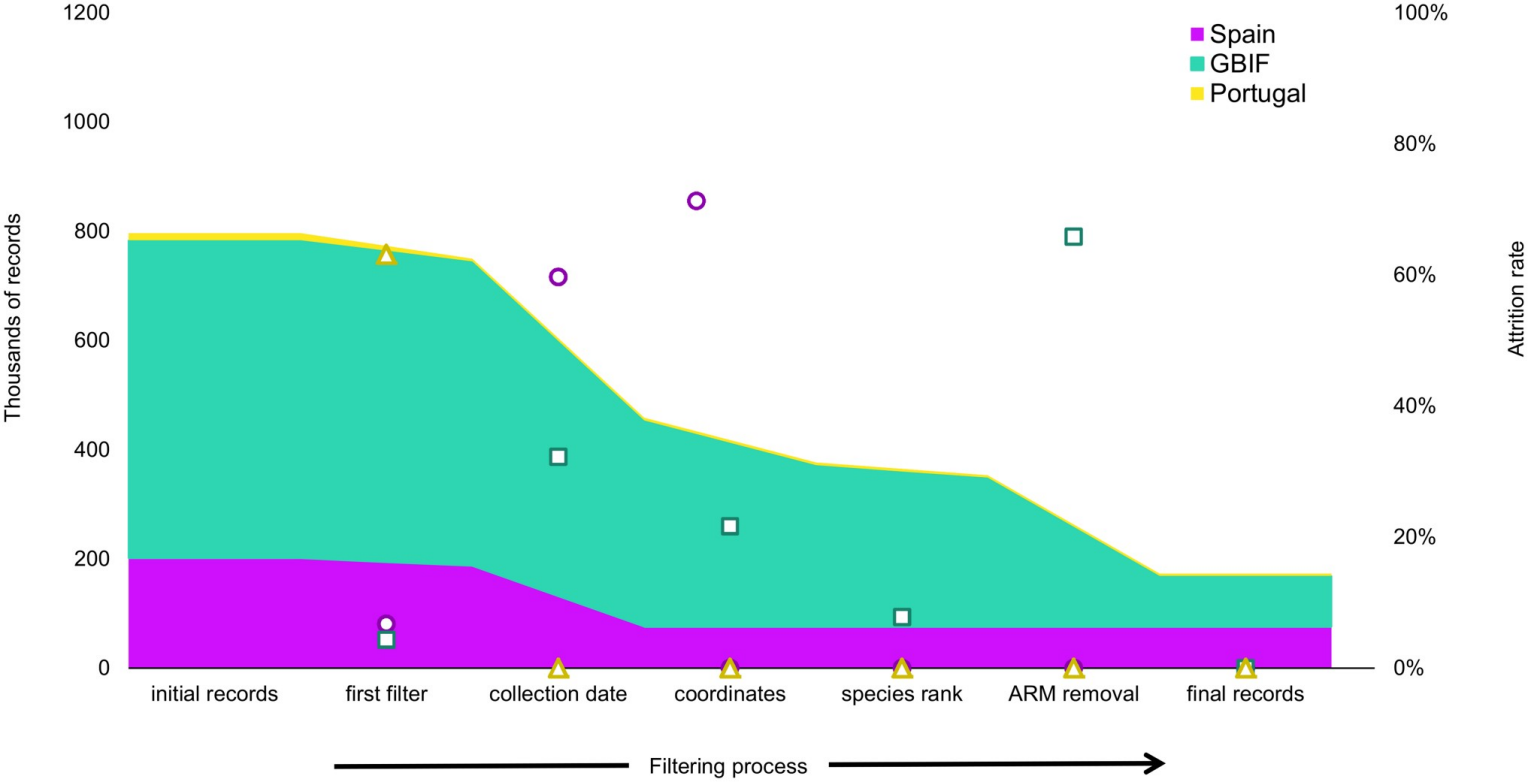
Number of records removed in each step of the filtering process for the three sources of data: Global Biodiversity Information Facility (GBIF), Spanish and Portuguese national atlases. Points show the leakage rate in each filtering step for each source of data. The first filter kept records from wild terrestrial mammals distributed in the Iberian Peninsula. The second filter removed records lacking collection date or species determination, and those having coordinates issues. ARM removal is the additional filtering step applied to the GBIF dataset to remove the data from the National Inventory of Terrestrial Species of Spain. The aggregation step collapsed the information by using the combination of scientific name, coordinates, and collection year.

582,720 records were retrieved from GBIF. We discarded 25,244 records from species not targeted for the study. Among the remaining records, 284,427 (48.81%) had quality issues such as lacking a collection date or species determination, or having wrong coordinates (Fig 1). Another large exclusion of data corresponded to the removal of the dataset from the National Inventory of Terrestrial Species of Spain that contained 179,757 records that had already been fed to GBIF at a different resolution. From the initial spatial coverage of 5,444 cells, after the filtering process, only 1,486 cells contained GBIF-mediated data (23.85% of the territory). The final number of GBIF records fit for this purpose was 93,292 (16% of the original dataset). Similarly, the data from the atlases yielded 213,563 records distributed in the Iberian Peninsula, but data quality issues precluded 126,713 of them (59,33%, Fig 1).

**Fig 2:**
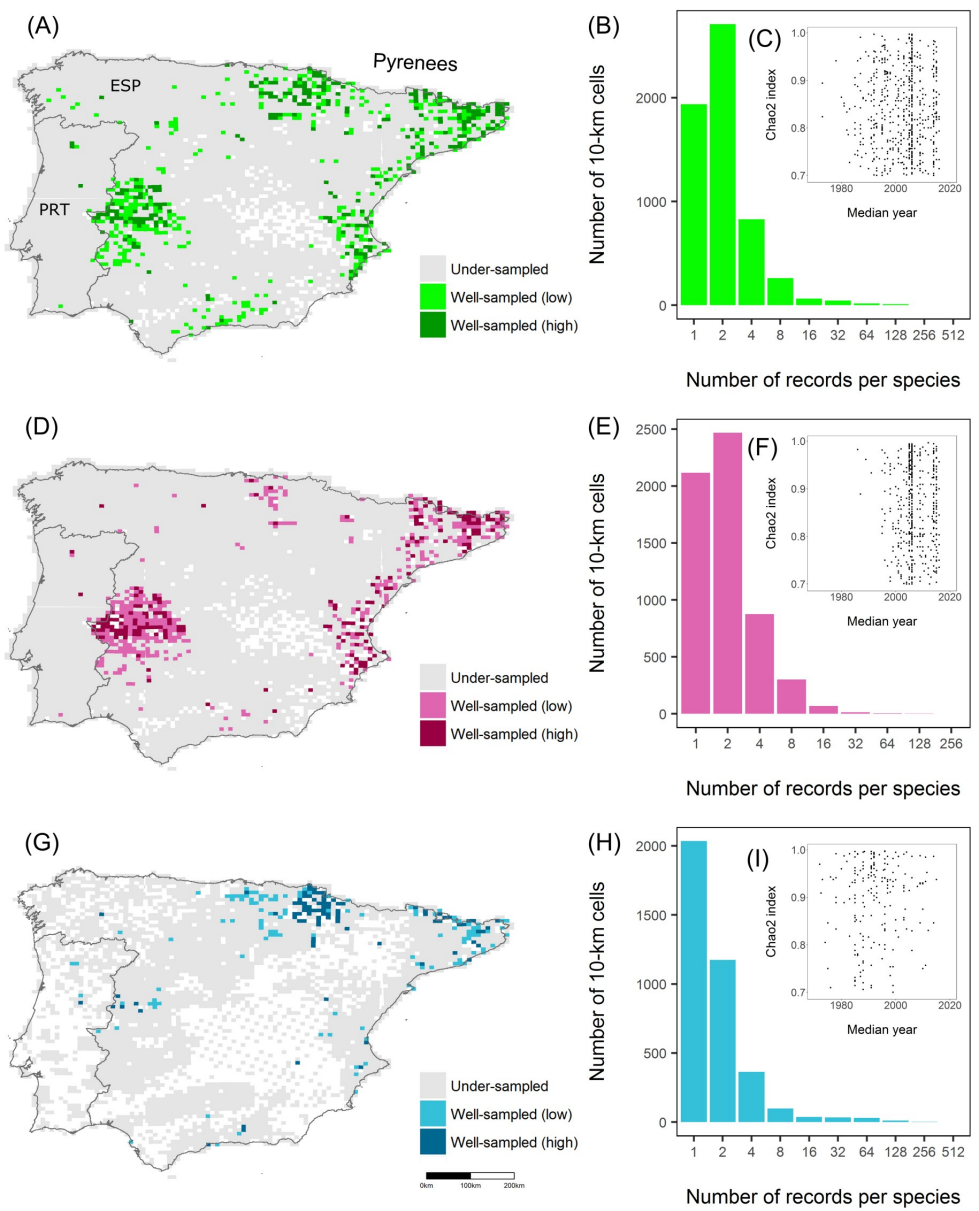
**Distribution of sampled 10-km cells across the Iberian Peninsula (PRT: Portugal, ESP: Spain) for (A) full dataset, (D) non-small mammals (Lagomorpha, Artiodactyla, Carnivora, Chiroptera and Erinaceidae), and (G) small mammals (Rodentia and Soricidae)**. Color shades highlight well-sampled cells according to the strict and lax criteria. Blank cells retained no data. The distribution of cells by their number of records per species (classes expressed as Preston octaves) is shown for the (B) full dataset, (E) non-small mammals, and (H) small mammals. The relationship between the Chao2 completeness index and the median year in which records were collected is shown for the (C) full dataset, (F) non-small mammals, and (I) small mammals. Records derived from the Global Biodiversity Information Facility and the Spanish and Portuguese national atlases.

Finally, the combination of both datasets resulted in a dataset with 179,767 records of 89 species distributed in 5,874 cells out of the 6,232 cells covering the Iberian Peninsula. Although the spatial coverage of mammals was high (Fig 2A), the number of records per species in each cell was generally low (mean: 1.37; 5^th^ and 95^th^ quantiles: 1, 4.89, Fig 2B). For example, 33% of the cells contained one record per species (Fig 2B). Concerning taxonomic groups, the analysis showed that non-small mammals contained 97,501 records of 55 species distributed in 5,850 cells, while small mammals (Rodentia and Soricidae) contained 82,266 records of 34 species found in 3,785 cells (Table 1).

**Table 1:** Summary of 10-km cells sampled across the Iberian Peninsula for full, non-small mammals (Lagomorpha, Artiodactyla, Carnivora, Chiroptera and Erinaceidae) and small mammals (Rodentia and Soricidae) datasets.

	Dataset		
	Full	Non-small mammals	Small mammals
Records	179,767	97,501	82,266
Observed richness	89	55	34
Mean C_c_	0.54	0.61	0.60
Mean C_m_	0.41	0.29	0.22
Sampled cells	5,874	5,850	3,785
Well-sampled cells (lax criterion)	620	531	205
Well-sampled cells (strict criterion)	262	184	89
Temporal range	1835–2018	1835–2018	1900–2018
Median years	2005	2006	1996

C_c_: completeness index based on Chao2 index

C_m_: completeness index based on the final SAC slope

The inventory completeness in the Iberian Peninsula was low for the full dataset. The mean value for C_c_ was 0.62 for all cells having at least 25 occurrences, the frequency threshold we allowed for a cell to be included in the calculations. Based on the lax criterion (n ≥ 25, C_c_ ≥ 0.7), 9.95% of the cells (620) were classified as well-sampled while the strict criterion (n ≥ 50, C_c_ ≥ 0.8) left 262 well-sampled cells (4.20%). We found that most of the well-sampled cells were located within Spain, particularly at both ends of the Pyrenees, on the Mediterranean coast and the midwest of Spain (Fig 2A).

### Environmental and temporal coverage

**Fig 3:**
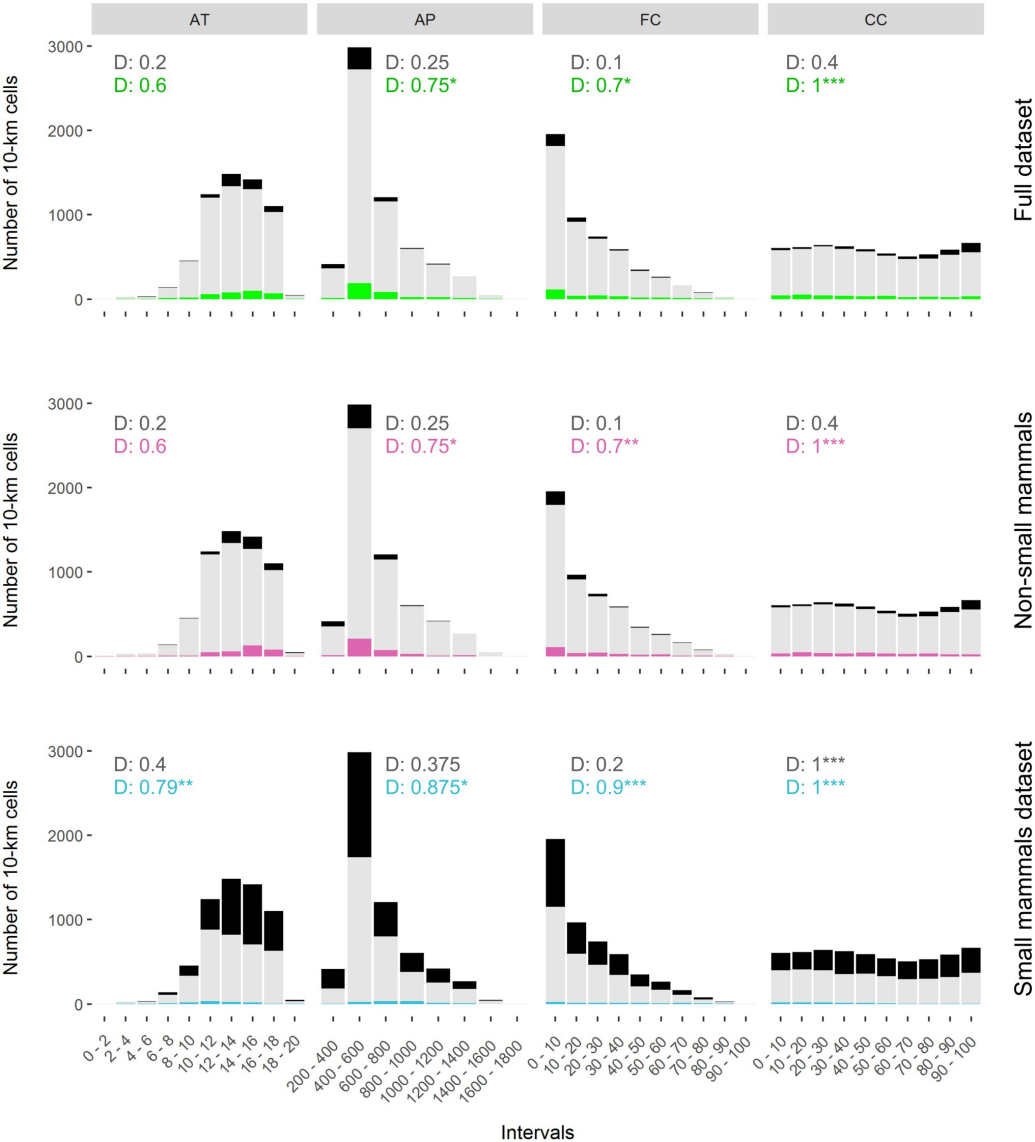
Survey coverage of well-sampled cells (number of records ≥ 25 and Cc ≥ 0.7) along four environmental variables: Annual Mean Temperature (AT), Annual Precipitation (AP), forest coverage (FC), and crops coverage (CC). Green: full dataset; pink: non-small mammals’ dataset (Lagomorpha, Artiodactyla, Carnivora, Chiroptera and Erinaceidae); blue: small mammals’ (Rodentia and Soricidae) dataset; grey: all cells having data (n = 5,874); black: rest of cells in the territory not holding data. Values correspond to the D-statistics from Kolmogorov-Smirnov goodness-of-fit tests. Lever of significance of 0.05 (*), 0.01 (**) and 0.001 (***).

The environmental coverage of well-sampled cells (lax criterion) was significantly low for all the selected variables (Fig 3). For the full dataset, the K-S D statistic was high, ranging from 0.6 to 1 (mean 0.76) in well-sampled cells. Similarly, poor environmental coverage of the well-sampled cells resulted for non-small mammals and small mammals (Rodentia and Soricidae) separately, with D values also ranging from 0.6 to 1 (mean 0.76 and 0.89 respectively, Fig 3).

**Fig 4:**
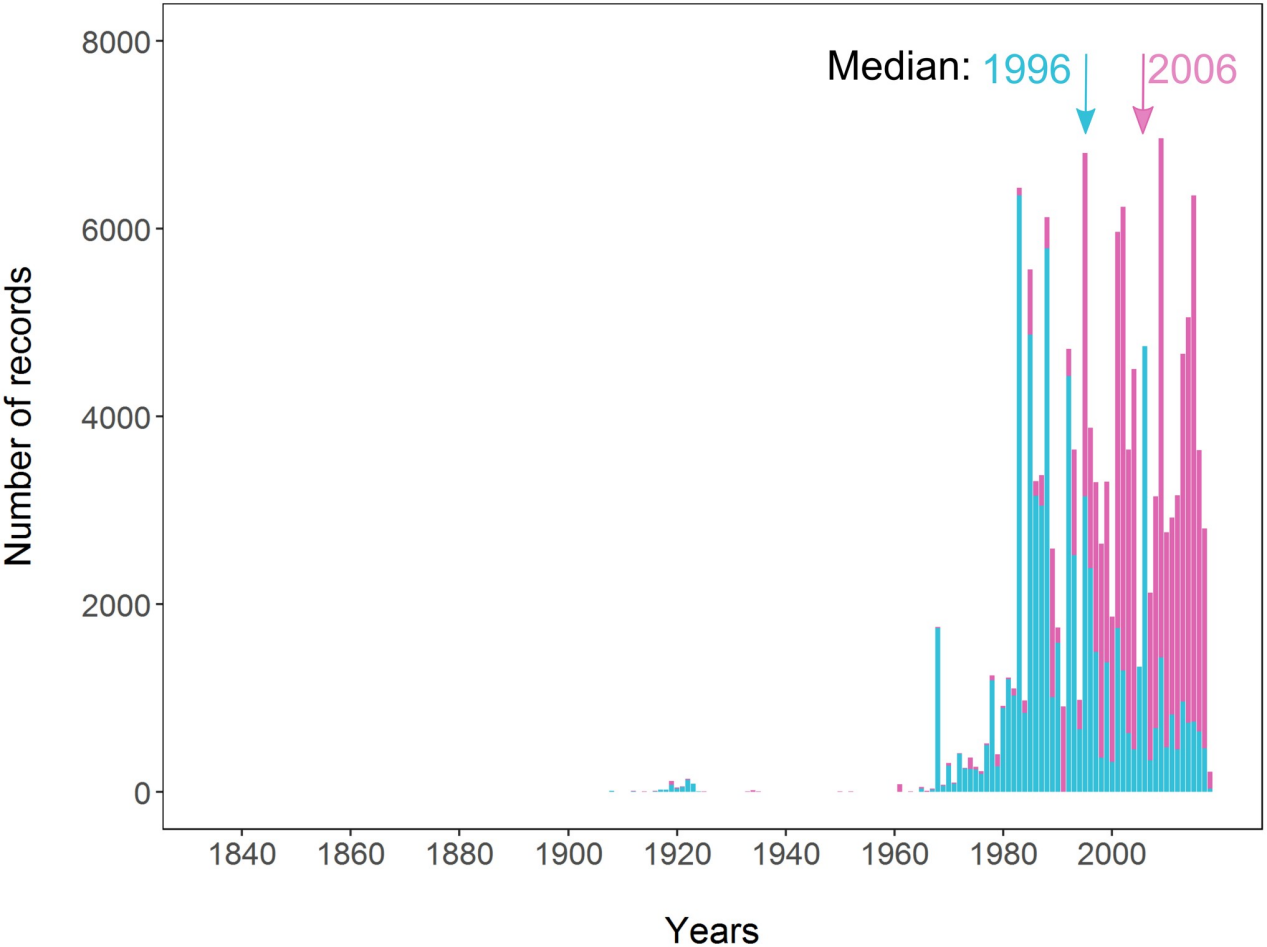
Temporal accumulation of records for non-small mammals (Lagomorpha, Artiodactyla, Carnivora, Chiroptera and Erinaceidae) in pink and small mammals (Rodentia and Soricidae) in blue. Arrows: year containing the data median for each series.

The temporal coverage of the complete dataset was 183 years, spanning from 1835 to 2018 (Fig 4). However, the inventory records were scarce from the early 1800’s to mid-1900’s with only 651 before 1960 for the whole Iberian Peninsula. Over the following decades records accumulated, peaking in 2006. There was a taxonomical difference in the accumulation pattern. Records in non-small mammals were mostly accumulated in the late 2000’s (median: 2006; p25: 2001; p75: 2012) whereas in small mammals (Rodentia and Soricidae) records accumulated about one decade earlier, in the 1990’s (median: 1996; p25: 1986; p75: 2006).

Over the full dataset, even though we found that the inventory completeness was negatively correlated with the median year in which occurrences were recorded (Spearman’s r = -0.06, p-value = 0.02), the effect was very small, and its significance was likely due to high sampling size. However, the separate taxonomic groups showed no significant correlation between the median collection year and the inventory completeness at α = 0.05.

**Fig 5:**
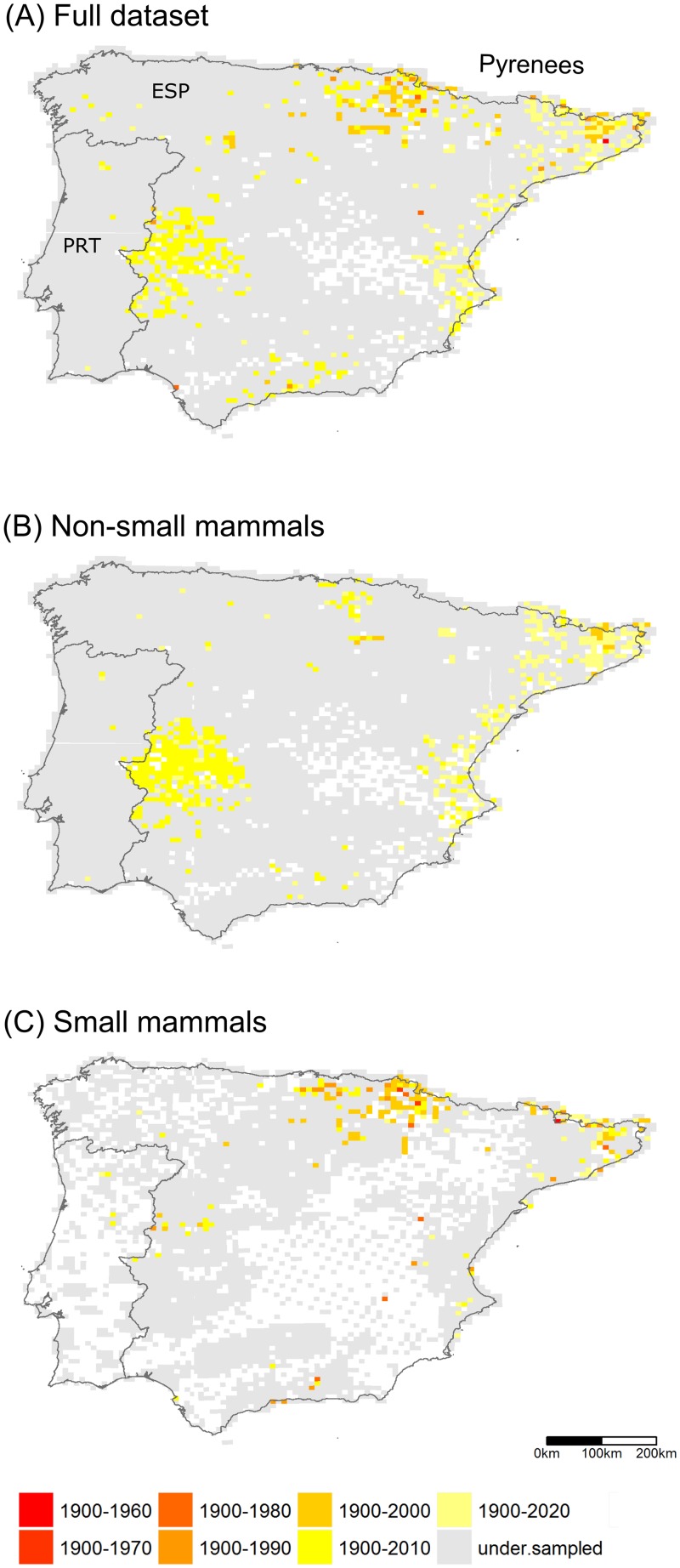
Recency maps of well-sampled cells (number of records ≥ 25 and C_c_ ≥ 0.7) across the Iberian Peninsula (PRT: Portugal, ESP: Spain) for the (a) full dataset and (b) non-small mammals (Lagomorpha, Artiodactyla, Carnivora, Chiroptera and Erinaceidae) and (c) small mammals (Rodentia and Soricidae) datasets. Yellow colors indicate cells that reached inventory completeness in recent decades. White cells have no data.

Nearby well-sampled cells (lax criterion) usually reached inventory completeness at roughly the same time (Moran’s I: 0.13, p < 0.001, Fig 5A). The pattern held after splitting the dataset by taxonomic groups (Fig 5B and 5C).

## Discussion

### Quality of DAK of terrestrial mammals in the Iberian Peninsula

Our goal was to characterize the availability of Digital Accessible Knowledge about terrestrial mammals distributed in the Iberian Peninsula, which could become suitable for further distributional, biogeographical or ecological studies requiring spatial and temporal representativeness. First, a coarse search in GBIF yielded more than a half-million occurrence data records from Spain and Portugal. Successive quality, suitability and reliability checks filtered out the majority of these data, and only 16.01% were deemed suitable for representativeness analysis on both the space and time dimensions. On the other hand, raw data collected from the national atlases yielded less than half as many records, of which 22.23% were fit for our purpose. Although the leakage rate [40] was thus higher in the case of the GBIF, overall, we observed comparable retention of records in both datasets as well as similar factors leading to attrition. The main reason for discarding records was data quality. While having partial data still allows records to be used for specific purposes, we needed complete records in our analysis. The lack of collection dates was the largest cause of leakage from GBIF and Spanish atlas data (Fig 1). The Portuguese atlas data had fewer data quality issues, but had a much larger proportion of records with inadequate distribution data that were removed first (Fig 1), and the collection dates were merely specified as before or after the year 2000.

The quality of data is of capital importance [41]. Imprecise data can suggest inaccurate diversity patterns [42] or biased species distribution models [43]. Errors such as misspelled species, or occurrences mismatching the expected territory for a record (e.g., political divisions) can be relatively easy to track and correct [44]. However, other errors such as wrong dates can easily go unnoticed and in many cases cannot be corrected although they could be detected (for example, misrepresenting unknown day of the month or month of the year [45], an anomalous concentration of records on the first day of each month or year). In our case, the coincidence of the publication date of the Spanish national atlas and a single batch of 179,757 observation records from 2007 found in the GBIF dataset led us to discover that those records had been assigned the atlas’ publication year rather than the actual occurrence dates. We were thus able to remove these GBIF records from the dataset, substituting them with the raw data. However, this type of detailed intervention may be difficult to accomplish when working with very large datasets, where these time-consuming checks for errors and inconsistencies [46] can easily become cost-ineffective. Thus, it is of utmost importance that data publishers provide accurate data and comprehensive metadata to inform data users about the true limitations of the data [40].

### Inventory completeness

Studies addressing inventory completeness are becoming more frequent [4,5,13,15,17,18,40,47–49]. Overall, these assessments tend to conclude that inventory completeness is spatially biased. We found that to be also the case for mammals in the Iberian Peninsula. Our analysis showed that DAK completeness was low, and spatially and temporally biased. Despite the relatively high proportion of territory cells holding data at the chosen spatial resolution, the number of records per cell was low (Fig 2B). The lax criterion excluded 90.05% of the cells from the full dataset. Well-sampled cells tended to be geographically aggregated, particularly in the western and eastern edges of the Pyrenees, the Mediterranean coast and one spot in the midwest of Spain (Fig 2A). Several causes might account for the comparatively high level of inventory in those areas, such as local atlases, research projects, or citizen science. When splitting the dataset by taxonomic groups, the spatial distribution of well-sampled cells for both groups did not substantially differ from the full dataset (Fig 2). DAK about non-small mammals seemed to be higher than that for small mammals in the Iberian Peninsula (e.g., 531 well-sampled cells compared to 205 using the lax criterion). Still, the overall completeness was very low.

Although both GBIF and the atlases yielded comparable numbers of records, as expected the spatial coverage provided by the atlas data was higher [50] (see Fig C in S1 Appendix). Grid-based biological atlases are often generated by compiling existing data, aggregating them to a reference grid that is reported as a single, generalized georeferencing for the data, and then seeking to fill in the cells lacking in data [51]. Consequently, atlases commonly represent trade-offs between their spatial coverage (which is generally high, and largely based on expert judgement) and the sampling effort (which is generally low). Also, the “unknown recurrence” (not reporting data considered as redundant) is common while constructing atlases [52]. All of this results in presence-only datasets with a low number of records and species per cell that may be assigned a low-resolution coordinate set (corresponding to the centerpoints of each cell in the grid). In our case, over the full dataset, almost half of the cells had equal numbers of records and observed species: that is, each species was reported as a single observation in the cell, probably leading to the underestimation of well-sampled cells [52].

### Survey and temporal coverage

Although the spatial coverage of mammals was high (i.e., most of the cells contained data, Fig 2), the low and spatially biased inventory completeness led to low, and spatially biased, survey coverage over all datasets (Figs 2A, 2B, 2C and 3).

The information about mammals in the Iberian Peninsula has been accumulating since the early 19th century, although most records that were made in, or converted into, digital form were only contributed in recent decades (Fig 4). We observed that records peaked at the start of the 21st century, but the inventorying activity decreased thence. One possible cause for the recent decrease in number of records might be the lag between data collection and data mobilization [53] although other causes could also be possible, such as an actual reduction in field campaigns or data collection, or possibly the processing of the backlog of natural history collections data [54]. The temporal pattern of inventorying was similar among taxonomic groups, even though they peaked at different times (Fig 4). The high inventorying of small mammals (Rodentia and Soricidae) during the later years of the 20th century in the western side of the Pyrenees (Figs 2G and 4) was driven by the publication of the extensive regional atlas for the province of Navarra [55]. Similarly, the publication of the Spanish national atlas resulted in a distinct increase in the number of records (Fig 4).

### Low completeness or low data sharing?

The results in this paper stress the necessity of mobilizing the data buried in personal databases, museums catalogs or research centers. The three countries in the Iberian Peninsula have been active in data sharing since GBIF was launched [45], with Spain leading the way with more than three-quarters of the peninsula’s data published as of September 2018 (https://www.gbif.org/occurrence/search?country=ES). Nonetheless, we are aware that many data may be still locked away [56,57]. Our results show not only a map of the inventory completeness of the mammals but also the areas where biodiversity data have been made digitally accessible. As the concentration areas tend to coincide with the political boundaries of a few, selected Spanish administrative divisions, it is quite likely that data exist as well in other regions within Spain that have not yet been mobilized through digital platforms and, thus, are not part of the DAK. However, unless scientific community and administrations incorporate the publication of data as a routine step in their workflow [58], access to data will continue to rely on the will of data holders to invest their resources to doing so.

Designing, surveying, and managing data have associated costs that can run high. Specifically, sampling mammals is a highly demanding task [31,32]. Mammal species usually show elusive behavior, or have nocturnal habits that influence their detectability and increase the efforts necessary to document them. Such effort involved in collecting data could be wasted if all the potential in the resulting data are not put to full use by properly converting them into DAK. We believe that releasing all the compiled information is a desirable and highly valuable step forward for research and conservation. As pointed out in several studies, data gathered in databases can be relevant for designing new and more efficient sampling protocols [59] even if the source of information is biased or scarce [11].

## Conclusion

Incomplete inventories limit the information yield, particularly when trying to determine the true spatial distribution of species as absences are difficult to confirm. Obtaining a better understanding of the species distributional and temporal patterns requires filling the existing information gaps, both positive and negative. While some gaps will surely be impossible to fill due to lack of surveys, much information from actual surveys may still remain to be discovered, debugged and published. Such data would allow us to fill historical gaps and to design better surveys to fill contemporary gaps. However, the volume of data by itself is not always a surrogate of increased knowledge [5,40]. We call for significant efforts to be made to increase the quality of the shared data, thus enlarging their fitness-for-use spectrum. The present and future of biodiversity research and conservation rely on freely available data. Good and sufficient data availability may facilitate better allocation of limited resources for research. With the release of that data we might be better prepared to understand changes in distributional patterns of species, and to answer questions when dealing with the biodiversity crisis we are facing.

## Supporting information

S1 Appendix Supporting information on methods. (PDF)

## References

[1] Rocchini D, Hortal J, Lengyel S, Lobo JM, Jimenez-Valverde A, Ricotta C, et al Accounting for uncertainty when mapping species distributions: The need for maps of ignorance. Prog Phys Geogr. 2011;35(2):211–26.

[2] Powney GD, Isaac NJB. Beyond maps: a review of the applications of biological records. Biol J Linn Soc. 2015;115(3):532–42.

[3] Sullivan BL, Phillips T, Dayer AA, Wood CL, Farnsworth A, Iliff MJ, et al Using open access observational data for conservation action: A case study for birds. Biol Conserv. Elsevier Ltd; 2017;208:5–14.

[4] Sousa-Baena MS, Garcia LC, Peterson AT. Completeness of digital accessible knowledge of the plants of Brazil and priorities for survey and inventory. Brotons L, editor. Divers Distrib. 2014;20(4):369–81.

[5] Meyer C, Weigelt P, Kreft H, Lambers JHR. Multidimensional biases, gaps and uncertainties in global plant occurrence information. Ecol Lett. 2016;19(8):992–1006. 10.1111/ele.12624 27250865

[6] Yesson C, Brewer PW, Sutton T, Caithness N, Pahwa JS, Burgess M, et al How Global Is the Global Biodiversity Information Facility? PLoS One. 2007;2(11):e1124 10.1371/journal.pone.0001124 17987112 PMC2043490

[7] Boakes EH, McGowan PJK, Fuller RA, Chang-qing D, Clark NE, O’Connor K, et al Distorted Views of Biodiversity: Spatial and Temporal Bias in Species Occurrence Data. PLoS Biol. 2010;8(6):e1000385 10.1371/journal.pbio.1000385 20532234 PMC2879389

[8] Troia MJ, McManamay RA. Completeness and coverage of open-access freshwater fish distribution data in the United States. Divers Distrib. 2017;23(12):1482–98.

[9] Hortal J, Lobo JM, Jiménez-Valverde A. Limitations of biodiversity databases: Case study on seed-plant diversity in Tenerife, Canary Islands. Conserv Biol. 2007;21(3):853–63. 10.1111/j.1523-1739.2007.00686.x 17531062

[10] Chao A, Jost L. Coverage-based rarefaction and extrapolation: Standardizing samples by completeness rather than size. Ecology. 2012;93(12):2533–47. 23431585 10.1890/11-1952.1

[11] Sánchez-Fernández D, Lobo JM, Abellán P, Millán A. How to identify future sampling areas when information is biased and scarce: An example using predictive models for species richness of Iberian water beetles. J Nat Conserv. 2011;19(1):54–9.

[12] Ariño AH, Chavan VS, Otegui J. Best Practice Guide for Data Gap Analysis for Biodiversity Stakeholders. GBIF Secretariat. GBIF Secretariat; 2016. 1–41 p.

[13] Idohou R, Ariño AH, Assogbadjo A, Glele Kakai R, Sinsin B. Diversity of Wild Palms (Arecaceae) in the Republic of Benin: Finding the Gaps in the National Inventory Combining Field and Digital Accessible Knowledge. Biodivers Informatics. 2015;10(2):45–55.

[14] Stropp J, Ladle RJ, M. Malhado AC, Hortal J, Gaffuri J, H. Temperley W, et al Mapping ignorance: 300 years of collecting flowering plants in Africa. Glob Ecol Biogeogr. 2016;25(9):1085–96.

[15] Yang W, Ma K, Kreft H. Geographical sampling bias in a large distributional database and its effects on species richness-environment models. J Biogeogr. 2013;40(8):1415–26.

[16] Soberón J, Jiménez R, Golubov J, Koleff P. Assessing completeness of biodiversity databases at different spatial scales. Ecography (Cop). 2007;30(1):152–60.

[17] Troia MJ, McManamay RA. Filling in the GAPS: evaluating completeness and coverage of open-access biodiversity databases in the United States. Ecol Evol. 2016;6(14):4654–69. 10.1002/ece3.2225 27547303 PMC4979697

[18] Peterson AT, Navarro-Sigüenza AG, Martínez-Meyer E. Digital Accessible Knowledge and well-inventoried sites for birds in Mexico: baseline sites for measuring faunistic change. PeerJ. 2016;4:e2362 10.7717/peerj.2362 27651986 PMC5018663

[19] Isaac NJB, Pocock MJO. Bias and information in biological records. Biol J Linn Soc. 2015;115(3):522–31.

[20] Sastre P, Lobo JM. Taxonomist survey biases and the unveiling of biodiversity patterns. Biol Conserv. Elsevier Ltd; 2009;142(2):462–7.

[21] Escribano N, Ariño AH, Galicia D. Biodiversity data obsolescence and land uses changes. PeerJ. 2016;4:e2743 10.7717/peerj.2743 27994967 PMC5157196

[22] Tessarolo G, Ladle R, Rangel T, Hortal J. Temporal degradation of data limits biodiversity research. Ecol Evol. 2017;7(17):6863–70. 10.1002/ece3.3259 28904766 PMC5587493

[23] Ariño AH, Otegui J, Villarroya A, De Zabalza AP. Primary Biodiversity Data Records in the Pyrenees. Environ Eng Manag J. 2012;11(6):1059–75.

[24] Ladle R, Hortal J. Mapping species distributions: living with uncertainty. Front Biogeogr. 2013;5(1):4–6.

[25] Gómez A, Lunt DH. Refugia within refugia : patterns of phylogeographic concordance in the Iberian Peninsula. In: Phylogeography of suothern european refugia. 2006. p. 155–88.

[26] Rivas-Martínez S. Memoria del mapa de series de vegetación de España (1:400000). Madrid: ICONA; 1987.

[27] Palomo LJ, Gisbert J, Blanco JC. Atlas y libro rojo de los mamíferos terrestres de España. Palomo LJ, Gisbert J, Blanco JC, editors. Madrid: Dirección General para la Biodiversidad-SECEM-SECEMU; 2007 588 p.

[28] Bencatel J, Álvares F, Moura AE, Barbosa AM. Atlas de Mamíferos de Portugal. 1a. Évora; 2017. 256 p.

[29] Ponder WF, Carter GA, Flemons P, Chapman RR. Evaluation of museum collection data for use in biodiversity assessment. Conserv Biol. 2001;15(3):648–57.

[30] Feranec J, Jaffrain G, Soukup T, Hazeu G. Determining changes and flows in European landscapes 1990–2000 using CORINE land cover data. Appl Geogr. 2010;30(1):19–35.

[31] Lyra-Jorge MC, Ciocheti G, Pivello VR, Meirelles ST. Comparing methods for sampling large- and medium-sized mammals: Camera traps and track plots. Eur J Wildl Res. 2008;54(4):739–44.

[32] Torre I, Arrizabalaga A, Flaquer C. Three methods for assessing richness and composition of small mammal communities. J Mammal. 2004;85(3):524–30.

[33] Chao A. Estimating the population size for capture-recapture data with unequal catchability. Biometrics. 1987;43(4):783–91. 3427163

[34] Oksanen J, Blanchet FG, Friendly M, Kindt R, Legendre P, McGlinn D, et al. vegan: Community Ecology Package. R package version 2.4–1. 2016.

[35] Fick SE, Hijmans RJ. WorldClim 2: new 1-km spatial resolution climate surfaces for global land areas. Int J Climatol. 2017;37:4302–15.

[36] European Environmental Agency. Corine Land Cover guide [Internet]. 2016. p. 1–163. http://www.eea.europa.eu/data-and-maps/data

[37] Clifford P, Richardson S, Hémon D. Assessing the Significance of the Correlation between Two Spatial Processes. Biometrics. 1989;45:123–34. 2720048

[38] R Core Team. R: A language and environment for statistical computing. R Foundation for Statistical Computing Vienna; 2016.

[39] The Inkscape Team. Inkscape 0.91. 2017.

[40] Peterson AT, Asase A, Canhos D, de Souza S, Wieczorek J. Data Leakage and Loss in Biodiversity Informatics. Biodivers Data J. 2018;6:e26826. 10.3897/BDJ.6.e26826 PMC6235996 30473617

[41] Chapman AD. Principles of data quality, version 1.0. Report for the Global Biodiversity Information Facility. Copenhagen; 2005.

[42] Maldonado C, Molina CI, Zizka A, Persson C, Taylor CM, Albán J, et al Estimating species diversity and distribution in the era of Big Data: To what extent can we trust public databases? Glob Ecol Biogeogr. 2015;24(8):973–84. 10.1111/geb.12326 27656106 PMC5012125

[43] Kadmon R, Farber O, Danin A. Effect of roadside bias on the accuracy of predictive maps produced by bioclimatic models. Ecol Appl. 2004;14(2):401–13.

[44] Chapman AD. Principles and methods of data cleaning—Primary Species and Species-Occurrence Data, version 1.0. Report for the Global Biodiversity Information Facility. Copenhagen; 2005.

[45] Otegui J, Ariño AH, Encinas MA, Pando F. Assessing the Primary Data Hosted by the Spanish Node of the Global Biodiversity Information Facility (GBIF). PLoS One. 2013;8(1):e55144 10.1371/journal.pone.0055144 23372828 PMC3555939

[46] Williams PH, Margules CR, Hilbert DW. Data requirements and data sources for biodiversity priority area selection. J Biosci. 2002;27(2):327–38. 12177532 10.1007/BF02704963

[47] Pelayo-Villamil P, Guisande C, Vari RP, Manjarrés-Hernández A, García-Roselló E, González-Dacosta J, et al Global diversity patterns of freshwater fishes—Potential victims of their own success. Divers Distrib. 2015;21(3):345–56.

[48] Ballesteros-Mejia L, Kitching IJ, Jetz W, Nagel P, Beck J. Mapping the biodiversity of tropical insects: species richness and inventory completeness of African sphingid moths. Glob Ecol Biogeogr. 2013;22(5):586–95.

[49] Lobo JM, Romo H, García-Barros E. Identifying recorder-induced geographic bias in an Iberian butterfly database. Ecography (Cop). 2006;6:873–85.

[50] Aizpurua O, Paquet J-Y, Brotons L, Titeux N. Optimising long-term monitoring projects for species distribution modelling: how atlas data may help. Ecography (Cop). 2015;38(1):29–40.

[51] Robertson MP, Cumming GS, Erasmus BFN. Getting the most out of atlas data. Divers Distrib. 2010;16(3):363–75.

[52] Lobo JM, Hortal J, Yela JL, Millán A, Sánchez-Fernández D, García-Roselló E, et al KnowBR : An application to map the geographical variation of survey effort and identify well-surveyed areas from biodiversity databases. Ecol Indic. 2018;91:241–8.

[53] Gaiji S, Chavan VS, Ariño AH, Otegui J, Hobern D, Sood R, et al Content assessment of the primary biodiversity data published through GBIF network: status, callenges and potentials. Biodivers Informatics. 2013;8:94–172.

[54] Ariño AH. Putting your Finger upon the Simplest Data. In: Biodiversity Information Science and Standards. 2018.

[55] Escala C, Irurzun JC, Rueda A, Ariño AH. Atlas de los Insectívoros y Roedores de Navarra. Análisis biogeográfico. Ser Zool. 1997;25:1–79.

[56] Ariño AH. Approaches to estimating the universe of natural history collections data. Biodivers Informatics. 2010;7:81–92.

[57] Johnson KG, Brooks SJ, Fenberg PB, James KE, Glover AG, Lister AM, et al Climate change and biosphere response: unlocking the collections vault. Bioscience. 2011;61(2):147–53.

[58] Chavan VS, Penev L, Hobern D. Cultural Change in Data Publishing Is Essential. Bioscience. 2013 6;63(6):419–20.

[59] Mccabe JD, Anich NM, Brady RS, Zuckerberg B. Raising the bar for the next generation of biological atlases: using existing data to inform the design and implementation of atlas monitoring. Ibis (Lond 1859). 2018;160(3):528–41.

