# Supplementary material for: Completeness of Digital Accessible Knowledge (DAK) about terrestrial mammals in the Iberian Peninsula
Source: PLoS One. 2019 Mar 8;14(3):e0213542. doi: 10.1371/journal.pone.0213542 (PMC6407841; doi:10.1371/journal.pone.0213542)
Supplement: S1 Appendix — (PDF) [file pone.0213542.s001.pdf]

Figure A. Correspondence analysis (CA) of the mammals' records from the Global Biodiversity Information Facility and Spanish atlas. Grey triangles represent the order and black points the basis of record of the records. The first axis explained the 70.5% distribution of mammals' records. The CA revealed a bimodal pattern, where small mammals (Rodentia and Soricidae) were grouped against the rest taxon levels (Lagomorpha, Artiodactyla, Carnivora, Chiroptera and the family Erinaceidae).

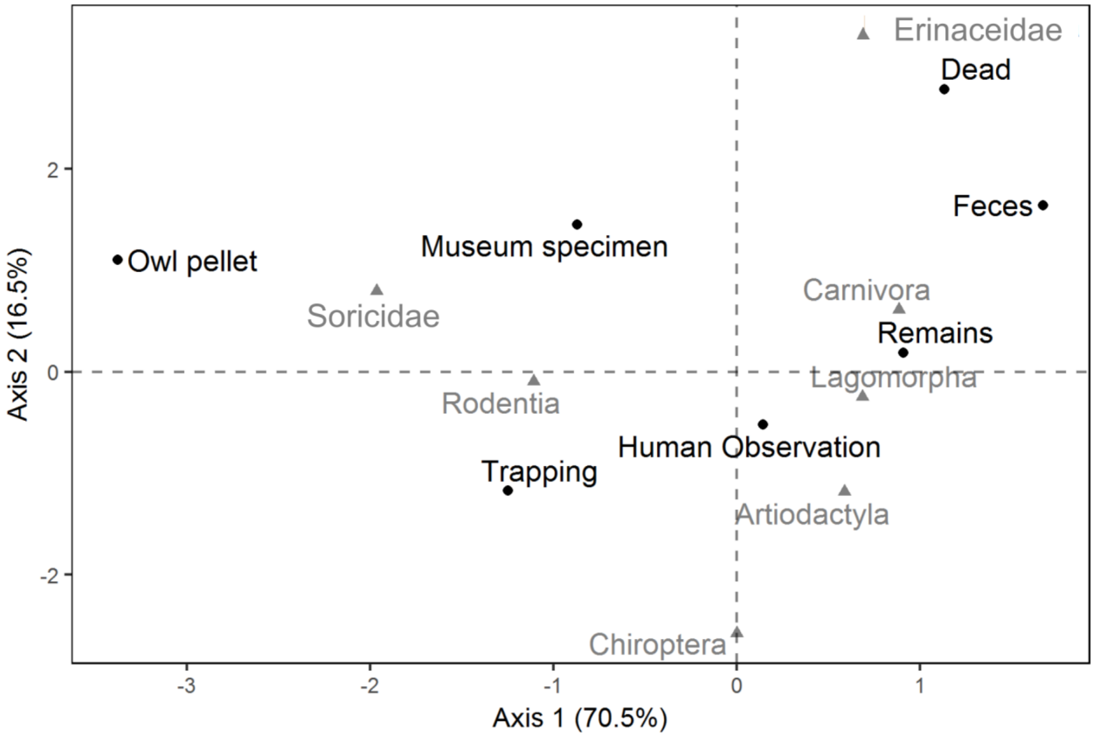

Figure B. Relationship between completeness based on the Chao2 index and the mean slope of the final 10% of the species accumulation curves for the full dataset and each taxonomic group. Lines represent the correlation.

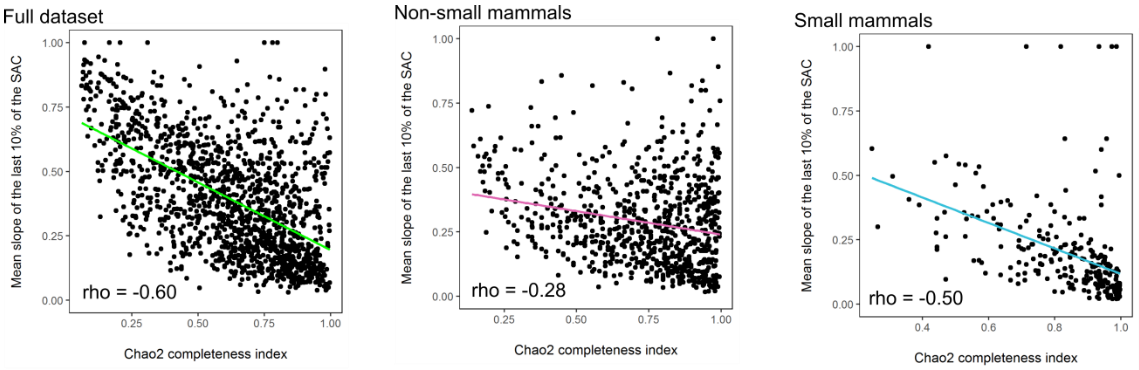

Figure C. Geographical distribution of the Global Biodiversity Information Facility and the Spanish and Portuguese atlases records before and after the clean-up process. Darker shades represent a higher number of records per cell. The color gradient of the scale is the same for all four maps.

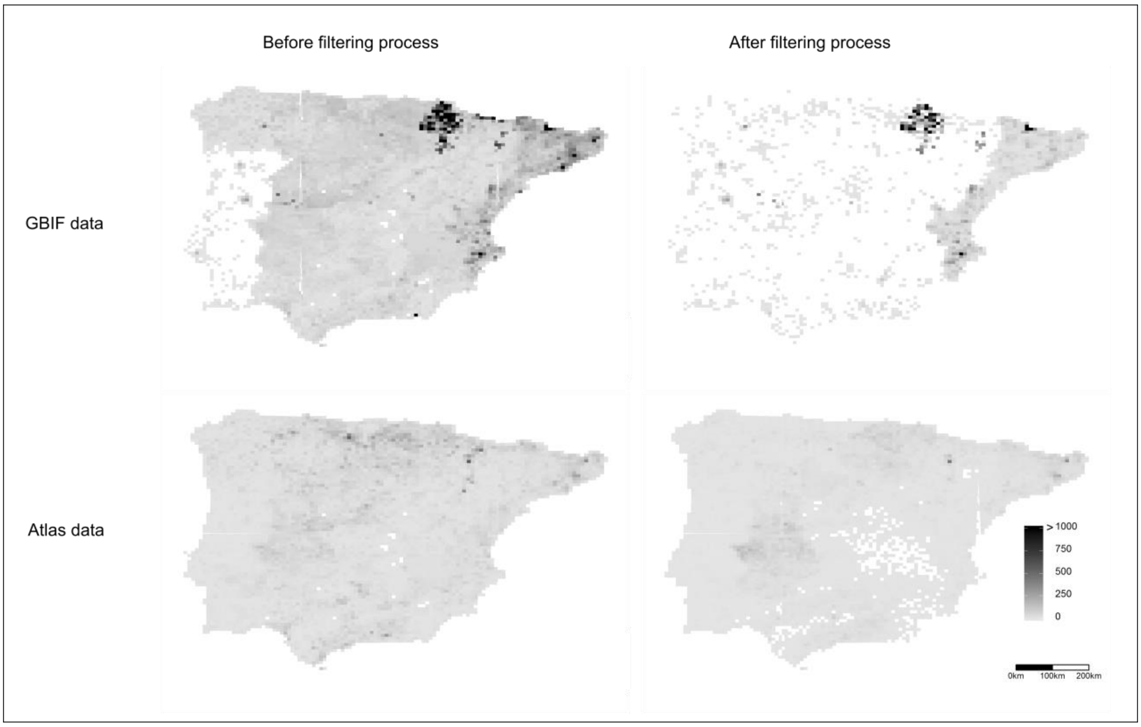

List A. References of the R packages used in this paper.

R Core Team. (2018). R: A Language and Environment for Statistical Computing. Vienna, Austria. Retrieved from https://www.r-project.org/

Heiberger, R. M. (2017). HH: Statistical Analysis and Data Display: Heiberger and Holland. Retrieved from https://cran.r-project.org/package=HH

Heiberger, R. M., & Robbins, N. B. (2014). Design of Diverging Stacked Bar Charts for Likert Scales and Other Applications. Journal of Statistical Software, 57(5), 1–32. Retrieved from http://www.jstatsoft.org/v57/i05/

Vallejos, R., Osorio, F., & Bevilacqua, M. (2018). Spatial Relationships Between Two Georeferenced Variables: with Applications in R. New York: Springer. Retrieved from http://srb2gv.mat.utfsm.cl

Dowle, M., & Srinivasan, A. (2018). data.table: Extension of `data.frame`. Retrieved from https://cran.r-project.org/package=data.table

Santos Baquero, O. (2017). ggsn: North Symbols and Scale Bars for Maps Created with “ggplot2” or “ggmap.” Retrieved from https://cran.r-project.org/package=ggsn

Heiberger, R. M., & Holland, B. (2015). Statistical Analysis and Data Display: An Intermediate Course with Examples in {R} (Second). Springer-Verlag, New York. Retrieved from http://www.springer.com/us/book/9781493921218

Wickham, H. (2011). The Split-Apply-Combine Strategy for Data Analysis. Journal of Statistical Software, 40(1), 1–29. Retrieved from http://www.jstatsoft.org/v40/i01/

Winston Chang. (2014). extrafont: Tools for using fonts. Retrieved from https://cran.r-project.org/package=extrafont

Bivand, R., Keitt, T., & Rowlingson, B. (2018). rgdal: Bindings for the “Geospatial” Data Abstraction Library. Retrieved from https://cran.r-project.org/package=rgdal

Bivand, R. S., Pebesma, E., & Gomez-Rubio, V. (2013). Applied spatial data analysis with R, Second edition. Springer, NY. Retrieved from http://www.asdar-book.org/

Oksanen, J., Blanchet, F. G., Friendly, M., Kindt, R., Legendre, P., McGlinn, D., … Wagner, H. (2018). vegan: Community Ecology Package. Retrieved from https://cran.r-project.org/package=vegan

Auguie, B. (2016). gridExtra: Miscellaneous Functions for “Grid” Graphics. Retrieved from https://cran.r-project.org/package=gridExtra

Paradis, E., Claude, J., & Strimmer, K. (2004). APE: analyses of phylogenetics and evolution in R language. Bioinformatics, 20, 289–290.

Francois, R. (2017). bibtex: Bibtex Parser. Retrieved from https://cran.r-project.org/package=bibtex

Fox, J., & Weisberg, S. (2011). An R Companion to Applied Regression (Second). Thousand Oaks {CA}: Sage. Retrieved from http://socserv.socsci.mcmaster.ca/jfox/Books/Companion

Wickham, H. (2009). ggplot2: Elegant Graphics for Data Analysis. Springer-Verlag New York. Retrieved from http://ggplot2.org

Hijmans, R. J. (2017). raster: Geographic Data Analysis and Modeling. Retrieved from https://cran.r-project.org/package=raster

Bivand, R., & Lewin-Koh, N. (2017). maptools: Tools for Reading and Handling Spatial Objects. Retrieved from https://cran.r-project.org/package=maptools

Warnes, G. R., Bolker, B., & Lumley, T. (2015). gtools: Various R Programming Tools. Retrieved from https://cran.r-project.org/package=gtools

Wickham, H., Francois, R., Henry, L., & Müller, K. (2017). dplyr: A Grammar of Data Manipulation. Retrieved from https://cran.r-project.org/package=dplyr

Bivand, R., & Rundel, C. (2017). rgeos: Interface to Geometry Engine - Open Source (GEOS). Retrieved from https://cran.r-project.org/package=rgeos

Neuwirth, E. (2014). RColorBrewer: ColorBrewer Palettes. Retrieved from https://cran.r-project.org/package=RColorBrewer

Pebesma, E. J., & Bivand, R. (2005). Classes and methods for spatial data in R. News, 5(2). Retrieved from https://cran.r-project.org/doc/Rnews/

Wickham, H. (2007). Reshaping Data with the reshape Package. Journal of Statistical Software, 21(12), 1–20. Retrieved from http://www.jstatsoft.org/v21/i12/
